# Walking Together: A Cross‐Cultural Study of the Relationship Between Religiosity and Substance Use Disorder

**DOI:** 10.1111/nhs.70418

**Published:** 2026-08-27

**Authors:** Camila Chagas, Leonardo Breno Martins, Tassiane Cristine Santos de Paula, Wellington Zangari, José Carlos Fernandes Galduróz

**Affiliations:** ^1^ Department of Psychobiology Universidade Federal de São Paulo‐UNIFESP São Paulo Brazil; ^2^ Department of Social Psychology Universidade de São Paulo‐USP São Paulo Brazil; ^3^ Department of Psychiatry Universidade Federal de São Paulo‐UNIFESP São Paulo Brazil

**Keywords:** homeless people, protective factors, religiosity, substance use disorder

## Abstract

The literature suggests that religiosity is a protective factor for substance use. However, individuals with substance use disorder (SUD) may actively cultivate their religiosity during substance use. This qualitative study analyzed the perceptions and meanings homeless people attribute to their substance use trajectories and religiosity, as well as the potential connections between these two themes. The analysis was conducted based on the content analysis technique proposed by Bardin. A total of 40 semi‐structured interviews were conducted with 20 Brazilian and 20 Portuguese participants. The present study found that religiosity can play a role in the substance use trajectory and the development of SUD by offering a subjective sense of protection, serving as a marker for the need for change, or providing relief from the suffering associated with SUD. We discuss how homeless people with SUD experience religiosity and how religious aspects traditionally seen as protective in the literature, such as church attendance, may paradoxically become risk factors for these socially vulnerable populations.

## Summary

- Religiosity can be part of the trajectory of drug use and the development of substance use disorders (SUDs).
- Homeless people with SUD in contexts such as harm reduction programs (Portugal) may have more accurate knowledge about drugs and be more critical of religious treatment institutions.
- Religiosity can bring a subjective feeling of protection, be a marker of the need for change, and, above all, can alleviate the suffering arising from the consequences of SUD.

## 1 Introduction

A number of reviews conducted across different decades suggest that religiosity is a protective factor against drug use in both young people (Donahue and Benson 1995; Kub and Solari‐Twadell 2013; Rew and Wong 2006; Russell et al. 2020) and adult populations (Buja et al. 2024; Chagas, Martins, Bezerra, et al. 2022; Chitwood et al. 2008; Khamis et al. 2022; King 1990; Koh et al. 2026), as well as an aid in the treatment of substance use disorders (SUDs) (Hai et al. 2019; Walton‐Moss et al. 2013). These findings have led to conclusions that religious approaches to the prevention and treatment of SUDs are “indispensable” (Grim and Grim 2019; Liu et al. 2025), in addition to the assertion that there is a “consensus” in the literature that religiosity is a protective factor in drug use (Algranti and Mosqueira 2018). However, such research, with many studies providing only a “snapshot” of a moment in an individual's life, may not consider aspects related to their life history as a whole, including their perceptions about their religious behaviors during substance use and other factors. Furthermore, an isolated interpretation of these studies might lead to the conclusion that religiosity plays an exclusively preventive role in maintaining abstinence from substances.

Recent research suggests that these findings may not represent the current state of the art, due to gaps identified in these studies (Chagas, Martins, Bezerra, et al. 2022; Chagas et al. 2024). A recent meta‐analysis that included 55 longitudinal studies showed that church attendance was the strongest protective variable in reducing the risk of alcohol and other drug use compared to other religious variables (Koh et al. 2026). However, none of the studies included in the review compared differences between atheists and religious people, with the protective results of religiosity being related to comparisons between the more and less religious. Moreover, some authors argue that the possible protective effects of religious attendance could be attributed to social control and the selection of a network of relationships that does not share drug use, making comparative analysis with secular activities that do not involve drug use necessary (Mellor and Freeborn 2011; Vogelsang and Lariscy 2020). A study comparing individuals who participated in religious or volunteer organizations found that both groups were more likely to be abstainers than those who did not participate in either activity (Kim et al. 2020).

Studies with exclusively religious samples, which analyze different profiles within the religious spectrum (e.g., high religiosity vs. low religiosity, Catholics vs. Evangelicals), are predominant in research on drug use (Jaeger et al. 2018; Kang et al. 2020; Karaca and Emre 2025; Koh et al. 2026; Russell et al. 2020; Torchalla et al. 2014). Other studies, even when comparing religious and non‐religious individuals, tend to group atheists, agnostics and those who are non‐religious together (Chagas, Martins, Bezerra, et al. 2022; Haber et al. 2012; Osuafor et al. 2016; Tuck et al. 2017). However, many non‐religious individuals who do not belong to any institutionalized religion still believe in a higher power or God (Chagas, Martins, Machado, et al. 2022; Tachi et al. 2020). This suggests that much of the current research on drug use and religiosity is grounded in the protective effects of high religiosity levels (Koh et al. 2026; Moscati and Mezuk 2014), attending churches, temples, or religious gatherings (Doolittle et al. 2021; Livne et al. 2021; Olashore et al. 2018) and affiliation with conservative religions (Guidolin et al. 2016; de Oliveira et al. 2020). Furthermore, there is evidence that the effect size of these studies tends to be very small or negligible (Buja et al. 2024; Chagas et al. 2024). Therefore, religiosity may be playing unexpected roles in people's lives, and the way research has been conducted thus far may not fully capture other aspects of this relationship.

The scarcity of qualitative studies on the relationship between drugs and religiosity underscores the lack of in‐depth exploration of the meanings and perceptions that individuals attribute to religiosity during substance use (Chagas et al. 2025; Drabble et al. 2016). People who use drugs and individuals with SUDs may be religious and maintain their belief during consumption, particularly when they live in countries where the majority of the population is religious, such as Brazil and Portugal, where around 90% of the population identifies as religious (IBGE 2010, 2022; Pew Research Center 2015). Therefore, the role of religiosity in the lives of individuals with SUD remains obscured by the paradigm of conflict between religiosity and drug use. A review analyzing the relationship between religiosity and harm reduction showed that most studies did not identify a conflict between these themes, indicating that religious beliefs can occur in harm reduction contexts related to drug use (Van Denend et al. 2025). Some qualitative studies, even though not specifically aiming to investigate this relationship, have provided data on the various religious beliefs and practices of individuals in places such as *Cracolândia* (Crackland in English), an area in downtown São Paulo, Brazil, known for its high concentration of open drug use (Adorno 2013; Trigo 2016, 2022; Fromm 2021) or in therapeutic communities (Nunes 2016; Williams 2016, 2017, 2020; IPEA 2017; Bloor et al. 2018). Another issue identified in these studies is the evangelization of individuals who use substances, with the primary goal of “taking them away” from this consumption through religiosity. This highlights the assumption that religiosity is not already part of the lives of individuals who use drugs.

Brazil and Portugal were chosen for the current study because, in addition to being predominantly religious, both have a high prevalence of substance use, especially alcohol, with studies indicating that almost half of the populations of the two countries used it in the previous year (Bastos et al. 2017; SICAD 2018). Despite these similarities, the religious landscapes of the two countries differ considerably. Although Catholicism remains the largest religion in both countries (IBGE 2010, 2022; Pew Research Center 2015), Brazil has experienced substantial growth in Evangelical Christianity in recent decades, resulting in a more evenly distributed religious composition between Catholics (50%) and Evangelicals (31%). Portugal, by contrast, has retained a strong Catholic majority (81%) (Pew Research Center 2015; US Department of State 2020). Despite the predominance of Christianity, Brazil is considered one of the countries with the greatest religious plurality, including a substantial number of individuals who report multiple religious affiliations (Chagas et al. 2024; R. Z. Coutinho and Golgher 2014).

Regarding drug use, in Portugal the consumption of all drugs was decriminalized in 2001 (Law No. 30/2000), and since then it has been considered a model for many countries around the world (Greenwald 2009; Neicun et al. 2019). In Brazil, although marijuana use was decriminalized in 2024 (Sousa and Barros Filho 2024), individuals who use drugs are still subject to criminal justice interventions, although imprisonment is not provided for in Law No. 11343/2006. Drug trafficking is the leading cause of incarceration in Brazil, accounting for approximately 30% of the total prison population, while in Portugal this percentage is 15% (SENAPPEN 2025). This is reflected in the differing positions of Brazil and Portugal in the 2021 Global Drug Policy Index, which assessed whether national policies adhered to the recommendations of the United Nations. Among the 30 countries evaluated, Portugal ranked 3rd, being considered one of the best countries, while Brazil ranked 30th, receiving the lowest rating (Thornton 2021).

Thus, the primary objective of this research is to understand the perceptions and meanings that individuals from Brazil and Portugal attribute to their own trajectories of drug use and religiosity, as well as how they connect (or do not connect) these themes. Furthermore, this study seeks to compare these two contexts through the accounts of homeless individuals with SUDs, given that examining these phenomena in marginalized contexts and extreme cases often provides more in‐depth or clearer insights into the issues being studied (James 2009).

## 2 Method

This is a qualitative study with samples of Brazilians and Portuguese individuals. The number of participants was established based on the theoretical saturation of the main themes under study. Saturation was reached when the meanings attributed to drug use and religiosity were repeated systematically until no new data emerged. The consolidated criteria for reporting qualitative studies (COREQ) were used to in this study (Tong et al. 2007). Informed consent was obtained from all participants and all interviews were recorded. This research was approved by the University's Research Ethics Committee (project no. 0931/2020‐CAAE: 36177620.7.0000.5505).

### 2.1 Study Design and Data Collection

Using purposive sampling, individuals experiencing homelessness in Brazil and Portugal who were receiving treatment for SUD were recruited. In Brazil, participants were recruited from a therapeutic community for the treatment of SUDs that serves homeless people in an inpatient setting. In Portugal, data collection was carried out at a shelter for homeless people that works in conjunction with harm reduction teams. Data collection took place in person in Brazil and Portugal in May 2022 and March 2023, respectively. The eligibility criteria were being in treatment for SUD and being 18 years of age or older.

Interviews took place in private rooms within the facilities and were conducted by two researchers trained to conduct the interviews. The first and second authors conducted the interviews in Brazil, while only the first author conducted them in Portugal. The first author, who conducted the interviews in both countries, has a background in psychology and conducts research on alcohol and other drugs, harm reduction, and the intersection of atheism and religiosity. The second author, who conducted the interviews in Brazil, works in the field of social psychology, with an emphasis on research on religiosity and anomalous experiences. The semi‐structured questionnaire covered questions about the trajectory of substance use (e.g., age at which substance use began, types of substances, treatments undertaken, when substance use began to become frequent) and about religious trajectory (e.g., religious affiliation, religious upbringing, importance of religiosity, attendance at church/temple/religious meeting) and, finally, how these themes evolved over time within the life story of each participant. A semi‐structured interview guide was developed by the research team and is available in the Supporting Information. All interviews were audio‐recorded and subsequently transcribed and analyzed using NVivo software, which allows for the organization, coding, analysis, transcription, and management of qualitative data. During data management, all participants were assigned an identification code to anonymize their names.

### 2.2 Qualitative Analysis

In order to achieve theoretical saturation of the main themes addressed, data analysis was conducted from the first day of collection. Content analysis was performed according to the technique proposed by Bardin et al. (2000) in four stages. The first stage consisted of organizing the analysis through floating reading; a technique that involves familiarizing oneself with the interviews and available content. This stage allows for exploratory mapping of the interviews, allowing impressions and guidance to emerge. In the second stage, coding, the collected material was processed. Coding was based on excerpts of recurring themes, such as the meanings attributed to the onset of excessive drug use: “I am solely responsible for my drug use”. According to Bardin et al. (2000), coding based on themes (instead of words) consists of identifying motivations, meanings, attitudes and discovering the core themes around which the discourse is organized. The third stage, categorization, consisted of grouping recurring themes and elements. This stage aimed to present a representation of the data and was carried out inductively (i.e., the categories emerged from the content found in the interviews). At the end of this phase, titles were created to represent the information apparatus with similar characteristics. The fourth stage, called inference, allowed for reflection and comparison between contexts through the interpretation of meanings contained in the interviews. The qualitative analyses were subjected to pairwise triangulation, in which the two researchers who conducted the semi‐structured interviews performed the categorizations. Any divergences were discussed with a third researcher.

## 3 Results

We conducted 40 individual semi‐structured interviews with people with SUD (20 Brazilians and 20 Portuguese). In Brazil, most respondents (60%) were under 40 years old, with an age range of between 21 and 60 years old. In Portugal, most respondents (85%) were over 40 years old, with an age range of between 33 and 65 years old. In Brazil, the largest religious group was evangelicals (40%) and in Portugal it was Catholics (50%). In Portugal, the second largest category was of people with no religion (40%), while in Brazil only two participants declared themselves to be without religion. Regarding drug use, in Brazil and Portugal most participants (80% and 95% respectively) used more than one substance together. However, in Brazil the most common combination was alcohol and cocaine, while in Portugal it was heroin and cocaine. Table 1 shows the characteristics of the participants.

**TABLE 1:** Characteristics of the Brazilian and Portuguese samples.

Characteristic	Brazil	Portugal
	*N* (%)	*N* (%)
Total	20 (100%)	20 (100%)
Gender		
Male	14 (70%)	15 (75%)
Female	6 (30%)	5 (25%)
Age		
20–29	7 (35%)	—
30–39	5 (25%)	3 (15%)
40–49	2 (10%)	5 (25%)
50–59	5 (25%)	5 (25%)
60–69	1 (5%)	7 (35%)
Substance used		
Cocaine	16 (80%)	18 (90%)
Cannabis	5 (25%)	3 (15%)
Alcohol	13 (65%)	4 (20%)
Tobacco	15 (75%)	14 (70%)
Heroin	—	13 (65%)
Polysubstance use		
Yes	16 (80%)	19 (95%)
No	4 (20%)	1 (5%)
Religion		
Catholic	3 (15%)	10 (50%)
Evangelical	8 (40%)	1 (5%)
No religion	2 (10%)	8 (40%)
Other religions	2 (10%)	1 (5%)
Multiple affiliations	5 (25%)	—

**FIGURE 1:**
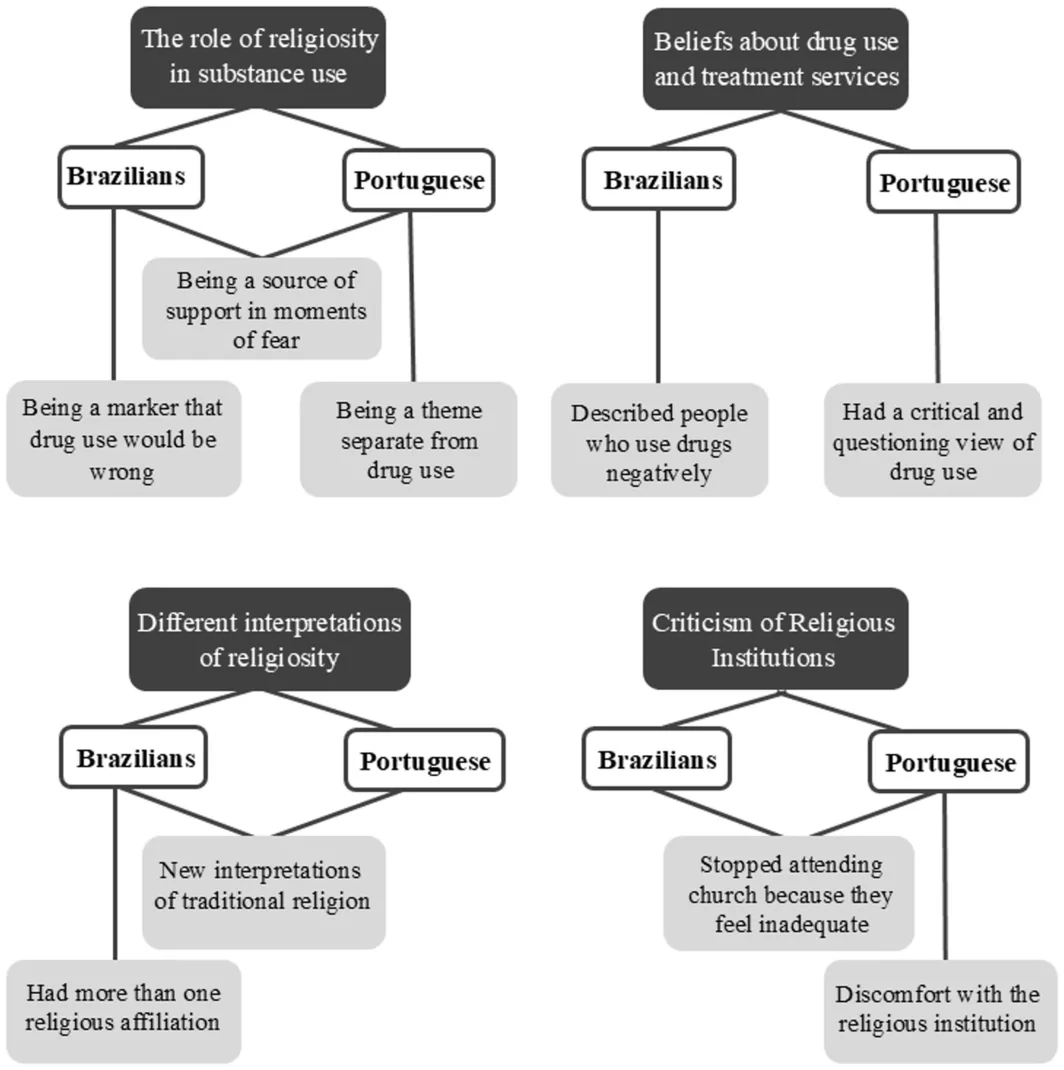
Categories of qualitative analysis and the comparison between Brazilians and Portuguese.

We identified four main overlapping categories in discourses on religiosity and drug use: the role of religiosity in substance use; from substance beliefs to religious beliefs; different interpretations and adaptations of religiosity; and criticism of religious institutions. Each main category presented differences and similarities in Brazil and Portugal, as illustrated in Figure 1.

### 3.1 The Role of Religiosity in Substance Use

For most of the Brazilian and Portuguese participants, religiosity played an active role during drug use. Religiosity was present in various areas of the subjects' lives, including during moments of drug use. Religious beliefs had at least three distinct roles: being a source of support in moments of fear about the consequences of drug use itself, being a marker that drug use would be wrong considering religious principles, or being intrinsically separate from drug use, with no apparent conflicts. However, only the first protective dimension of religiosity was observed in both countries. In Brazil and Portugal, participants reported feeling certain that they were being cared for and protected by God. Furthermore, religiosity in both countries seemed to be an integral part of the context of substance use. The majority of Portuguese participants felt protected by their belief in God from the possibility of overdose or contracting diseases, expressing the certainty that nothing bad could happen, even if they engaged in risky behavior, as described by some participants.

I've always trusted in God. I think God told me it wasn't my time yet. I've done so many stupid things with alcohol, drugs, and everything else, but God has always been there. My family always says, ‘How are you still here?’ They don't drink, use drugs, or anything like that, and they're always in and out of hospital. Meanwhile, I've done everything: alcohol, drugs, pills, you name it and I'm still here. There aren't many people like me. Very few (Portuguese participant 26).

The deities are very strong and even when [I am] using drugs, the entities influence. They appear. I have mediumship. I see, I feel. They influenced me and him [my boyfriend] to stop. Knowing that they are there watching the wrong things we are doing. Do you understand? And that was what I talked to him about the most. The times we were wasting money for nothing. Do you understand? Even in the precarious conditions we were in when we were on the streets. The entities were there. Even in those moments I asked them to help me, and they helped me. It confirmed it. They helped me, but for me to have the mind to do the right thing and at the time I did the wrong thing and used drugs (Brazilian participant 1).

God is always present, with or without drugs. When I'm going through withdrawal I say ‘oh my God help me’, ‘Help me make money’, ‘Help me stop the hangover’ (Portuguese participant 33).

In the Brazilian context, the fear of death as a consequence of drug use appeared as a subjective feeling that one was doing something wrong, but that God would still be present. An illustration of this can be seen in the following interview extracts.

It was only when I was using drugs too much that I got that feeling‐man, I think I'm going to die. Only then I thought about God. The next day, I was already using drugs again (Brazilian participant 17).

I used to think about God a lot. I knew that what I was doing was wrong. I knew that God could hold me accountable for what I was doing, because I was really destroying myself. I used to think about that a lot, about what I was doing. I used to think about it more when I was high on drugs. When I thought I was going to die, I would pray to God a lot, and that would bring me closer, you know? (Brazilian participant 14).

In the Portuguese sample, two participants felt very differently and believed that the religious and drug use dimensions should be considered separately. This view is clearly portrayed by Portuguese participant 29:

You shouldn't mix things up. I think there should be respect in everything and doing this [using drugs] with religion is disrespectful. I can't even think about that. In fact, I can't think about one thing and the other at the same time. They are separate things and they shouldn't be mixed up, in my opinion.

### 3.2 Beliefs About Drug Use and Treatment Services

Beliefs about substance use, treatment, and the characteristics of people who use drugs differed between Brazilian and Portuguese participants. While Brazilian participants described people who use drugs negatively and stereotypically, Portuguese participants tended to have a critical and questioning view of drug use and treatment models, especially in therapeutic communities. The following statement illustrates the opinion of some Brazilian participants about drug use:

Because the addict's problem is to blame someone else. So, like, the addict has this problem of wanting to blame someone else, but the problem is him and not the other person. So here you learn that the problem is not the other person, the problem is you. He wants to use drugs no matter what, so he must find an excuse to use drugs and that's the problem (Brazilian participant 10).

The negative symbolic charge of the person who uses drugs was repeated by many Brazilian participants and seems to be reaffirmed by the professionals who work in the treatment of SUD, for example, Brazilian participant 6 reported that “they say that we sabotage, invent things, so we are not sure if it is that or not. Only someone who has studied can know in depth”.

For the Portuguese participants, the main criticisms of therapeutic communities were: the 12‐step model, religious basis, labor therapy, lack of in‐depth analysis of practical day‐to‐day issues and detoxification without medication. Two statements by Portuguese participants are illustrative examples of the criticisms of the 12 steps.

The other one was the 12 steps, it was about following them without criticism. What I realized was that we didn't get past the first step, which is admit, admit, admit. I've already admitted it, so let's move on (Portuguese participant 22).

And they only dedicate themselves to the 12 steps and I don't think it's really ethical, but you end up learning something about the 12 steps that you don't know about yourself yet. How are you going to learn about the 12‐step program if you don't really know yourself inside? Why things happened, the mistakes you made in your life, the reasons that led you to do this or that (Portuguese participant 30).

Reports on forms of treatment may also be the result of a collective perception that goes beyond direct experience in such places. In other words, while some Portuguese participants reflected on their own experience in therapeutic communities, others expressed reservations based on the experiences of other people, for example, in the report of Portuguese participant 32:

The only therapeutic communities I know of… but for me this is a farce, it's a way to make money. Because they accept patients there, they work in the fields for them for free, the detoxification is cold, they force the person to practice, to be Catholic. Even if you're not, you must be Catholic, you must go to mass. This is what many people tell us, I've never been there but I've heard stories and then we go around here collecting items for them, but they do a business, they collect and resell. That's all. You work for them for free and that's why I don't believe in it.

The Brazilian participants, in addition to not expressing criticism of treatment institutions, presented other beliefs such as the “escalation of drug use”, stating that individuals start by using marijuana and are gradually introduced to the consumption of so‐called “hard” drugs, such as crack. Brazilian participant 10 stated “Marijuana, the first drug I tried. All drug addicts start with marijuana and move on to cocaine.”

### 3.3 Different Interpretations of Religiosity

As for religious beliefs, despite contextual differences such as the predominance of Catholics in the Portuguese sample and Evangelicals in the Brazilian sample, the interpretation of religiosity itself in both countries involved new interpretations of traditional religion (Chagas et al. 2024). For example, in both countries, some participants, even though they declared themselves to be Evangelical or Catholic, gave explanations that differed from religious doctrine, for example, believing in reincarnation.

Because I believe more in reincarnation, even in the Congregation my spiritual side was like that. I go there, I like it there, but because of the spiritual side and not because of the personal side. Everyone has their own way of interpreting it. So even in the Congregation I always believed in that. I always believed that because I had three overdoses and haven't died yet it's because my mission here on earth isn't over and if I'm here today it's because I have a path to follow (Brazilian participant 20).

I was raised Catholic and I occasionally went to spiritism. And I always liked and identified a lot with spiritism. Both Mesa Branca and Umbanda. The doctrine is different, but the end is the same (Brazilian participant 3).

I believe in something, but not exactly in a religion. I believe that there is a force, that there is something, but I don't know how to express what it is. I always try to hold on to something, yes. I even have my faith in Our Lady of Fatima, I have my faith in Shiva. I cling a little to everything. I try to find what I believe in within what I am learning (Portuguese participant 24).

However, only the Brazilian participants declared themselves as belonging to more than one religion at the same time, with five of them classified as having multiple religions (Table 1), such as “evangelical and Candomblé practitioner” and “Catholic, Spiritist and Umbanda practitioner.”

### 3.4 Criticism of Religious Institutions

Despite being religious, some of the participants were critical of established religions. Six of the Portuguese expressed their discomfort with religious institutions, especially the Catholic Church, which is predominant in Portugal. The criticism included the reports of pedophilia among priests, the economic ambition of the church, the loss of biblical essence and teachings, and the manipulation of people. Such issues resulted in some distancing and aversion to churches, as illustrated by some participants.

Look at what's happening now with pedophile priests, the church, the church, the church is the biggest lobby of the biggest lobbies in the world. They manipulate people, it's crazy. All religion is about is manipulating people and making money and profiting from it. For me, that's how I think, whatever religion is. And it's men who manipulate that. They're people like us. And human beings are greedy by nature, by the way (Portuguese participant 32).

I don't believe in the church as an entity. Personally, I think it's just a business. It buys your sins, it buys your little place in heaven, it buys everything. It's a business. I don't see that there's really any faith, but I buy the faith. It doesn't make sense. It loses its essence and stops making a little sense (Portuguese Participant 24).

Other Portuguese participants did not feel comfortable within a religious environment, such as a church, which was related to both the perception that drug use is not appropriate in that environment, and the perception that their own appearance (inappropriate and/or dirty clothing) was not accepted in those places. This can be illustrated by excerpts of the accounts of two participants.

At 16, 17 years old, but because of pressure from my grandmother and mother to go to mass, to church. Later, in a way, I went because it made me feel good, going to mass, praying. So going there made me feel good. But with consumption, habits change and I started to feel uncomfortable with church. It happened that I was using and at that time if I saw a church I would even go in and be there, but when the consumption got worse I stopped going to church (Portuguese participant 22).

No, I went because my family insisted. I am a believer, but I don't just go to church. I don't like the bigots who gossip. People ruin the church (Portuguese participant 27).

Although some participants expressed their reasons for feeling uncomfortable with the church, others were less explicit, stating, for example, that the church “bothered” them. Among Brazilian participants, there was no explicit criticism of the church as an institution, although some reported that they no longer attended. Furthermore, some participants reported the need to make an effort to learn about religions that were not originally their own in order to participate in certain religious rituals within the therapeutic community, as illustrated in the examples below:

In 2011 I joined Candomblé, in 2011 I had my head shaved for my orixá, but I distanced myself a bit. Even so, I'm still part of Candomblé, but joining here I also had to become an evangelical Christian to fit in. I believe in nature and in God (Brazilian participant 2)

They ask me: What praise song do you want to hear? I say, “I don't know, I'm not familiar with it.” Now, if you mention something about Axé, Axé Bahia, African Axé, an oríkì, a bômbata [a rhythm, chant, or ritual expression of the Afro‐Brazilian church called Candomblé], then people get all worked up, but I'm trying to learn the gospel songs (Brazilian participant 1).

## 4 Discussion

This study extends the current understanding of the role of religiosity in the lives of people with SUDs in several ways. It suggests that religiosity is not only related to protective factors for drug use and support during treatment but may also be part of the trajectory of drug use and the development of SUD. This study also highlights important differences between the two settings: In Portugal, homeless people with SUD recruited from harm reduction services tended to have more accurate knowledge about drugs and express greater criticism of religious treatment institutions, whereas in Brazil, participants undergoing treatment in a religious therapeutic community demonstrated more stigmatizing beliefs about drugs. Finally, our findings suggest that religiosity may provide a subjective feeling of protection, serve as a marker of the need for change and, above all, help alleviate the suffering associated with SUD.

The use of substances, especially illicit ones, does not seem to be associated with decreased religiosity. Similar results have previously been found in qualitative studies with homeless Italians (Testoni et al. 2020) and with African‐American women who used cocaine (Brown 2006), in which religiosity was an integral part of the context of drug use. Similarly, our study found that religiosity was present in the lives of participants before drug use and continued to be present during drug use and the progression to a SUD.

The literature suggests that religiosity can be used as a system of meaning to cope with stressful situations, because in addition to providing explanations, it acts as a form of control and predictability (Ano and Vasconcelles 2005; Dolcos et al. 2021; Imran and Leng 2025; Koenig et al. 2019; Pargament et al. 2001; Pham et al. 2020; Puchalski et al. 2009; Shetty et al. 2025). In the present study, participants experienced stressful and extreme conditions, such as living on the streets and having a SUD, and coping through religiosity emerged in a reinterpreted form in these contexts. Although they tended to distance themselves from traditional forms of religious practice (such as attending church), religiosity was mainly used to alleviate or protect against the consequences of drug use (e.g., seeking God's help to improve hangovers or protection from communicable diseases). Thus, religiosity acted as a form of protection against the daily adversities experienced by people in marginalized situations.

In both contexts, instead of acting as a traditional protective factor (e.g., reducing the likelihood of substance use), religiosity tended to be a subjective protective factor in coping with the difficulties associated with substance use. In this context, subjective protection is defined as a subjective and profound sense of being protected from the adversities and negative consequences of drug‐related risk behaviors (e.g., overdoses) by a higher power or God. However, this subjective feeling of protection associated with religiosity can also be a risk factor, as it may lead individuals to expose themselves to riskier situations due to their belief in divine protection.

The role of subjective protection, therefore, would not be to reduce or increase drug use, but rather to function as a psychological resource through which individuals experiencing extreme social vulnerability sustain a sense of hope in a world that marginalizes them, and feel that an external divine agent protects them from everyday adversities. This form of protection appears to be independent of abstinence, as it may remain present unconditionally during drug use. This understanding of the role of religiosity is related to the vulnerable nature of the populations studied in both countries. Therefore, our results may differ from the literature which indicates protective health outcomes associated with high levels of religiosity (Koenig and Larson 2001; Pargament et al. 2004) or health risk factors associated with spiritual struggles or doubts (Faigin et al. 2014; Krause et al. 2018). Instead, our results may be closer to what Puchalski et al. (2009) understood as meaning in life, which includes, among other things, the relationship with God or a higher power. In the present study, this relationship with the divine may be simultaneously associated with both positive outcomes (e.g., feeling valued, cared for, and having a sense of purpose and meaning) and negative outcomes (e.g., exposure to risk behaviors), without one diminishing the intensity of the other. This suggests that the same religious belief may promote coping with adversity and a sense of control while also increasing vulnerability by altering risk perceptions related to drug use. These findings extend previous research showing that high levels of religiosity may play a temporary role in abstinence, further highlighting the complexity of religiosity in the lives of people who use drugs (Jaramillo et al. 2022; Yeterian et al. 2015). Furthermore, some research shows that homeless people may have different spiritual needs than people with fixed Housing (Webb et al. 2018), with religiosity acting as a way to “survive on the margins of society” (Mpofu 2021). Thus, examining the relationship between religiosity and drug use in greater depth revealed that subjective protection may operate across multiple domains of life affected by substance use, providing meaning, sustaining hope, and offering psychological resources for coping with the challenges of social marginalization.

Furthermore, none of the participants expressed doubts about the higher power in which they believed or negative feelings about their own religiosity. This finding differs from research showing that some stressful and distressing situations can foster spiritual struggles and doubts about one's own belief system, such as feelings of divine punishment or demonic influence (Fuentes‐Ferrada et al. 2023; Loewenthal et al. 2022; Pirutinsky 2024), and be a risk factor for negative health behaviors (Aggarwal et al. 2023; Pargament et al. 2004; Park et al. 2018; Pirutinsky 2024). However, although we found that religiosity acts as support and security in a daily life of challenges and uncertainties, it also favors a negative dimension of coping through greater exposure to risky behaviors. Future research can investigate this association between feeling protected and the chances of being exposed to riskier situations in more detail, using both qualitative and quantitative methods. These investigations could elucidate other difficulties arising from risk‐related substance use and what other roles religiosity might play in the lives of this population.

In respect of church attendance, although there is a significant body of research showing this to be a protective factor against the use of various drugs (Doolittle et al. 2021; Koh et al. 2026; Meyers et al. 2017; Olashore et al. 2018), in the present study, this practice was abandoned by all participants in both Brazil and Portugal. These participants, who tended to deviat from social norms of dress, posture, and practices, felt uncomfortable within the religious environment, and had negative perceptions of the church and found it unwelcoming. Similar findings were observed in another qualitative study in which drug‐using participants reported not feeling welcome in church (Szott 2020). This difficulty in accessing this population has already been observed in countries such as the United States and Mexico, where religious leaders initiated a movement called “Church Without Walls” to enable homeless people to attend church outside traditional buildings, to which they often have limited access (Carvalhaes 2016). Traditional religious places may represent acceptance for a portion of the general population and exclusion for those experiencing homelessness, mainly because this group tends not to fit the social norms advocated by religion. The literature describes how religious ostracism (i.e., being excluded and isolated in religious environments) can contribute to feelings of worthlessness, powerlessness, and invisibility, with lasting negative effects on mental health (Boon and Brown 2020; Hales et al. 2020). This topic remains underexplored in the literature with regard to homeless people with SUD, largely due to the difficulty of accessing this population, especially given that most research is conducted using online questionnaires among individuals with higher levels of education who are not in vulnerable situations.

The Portuguese participants criticized religious institutions, describing them as sites of corruption and financial extraction. Such criticism was observed only in the Portuguese sample, and may reflect the dominant position of the Catholic Church in Portugal, and the limited availability of alternative denominations, factors that may contribute to dissatisfaction with the Church (J. P. Coutinho 2023; Duque and Durán Vázquez 2020; Roussou 2015) and the growth of the number of individuals identifying as non‐religious, which constitutes the second largest population group in Portugal after Catholics (INE 2021). In Brazil, the diversity of the religious landscape, with its wide range of churches, temples, and rituals, makes it easier for individuals to seek alternatives when dissatisfied with their religious institution (Chagas, Martins, Machado, et al. 2022).

Although religiosity in the two samples showed surface‐level differences, they shared underlying similarities. In Brazil, five participants identified with multiple religious affiliations (e.g., the same participant identifying as both Evangelical and a Candomblé practitioner), a phenomenon referred to as intrapersonal religious pluralism (Chagas et al. 2024; Chagas, Martins, Machado, et al. 2022; Peres et al. 2020). In Portugal, this blending of religions emerged through participants' narratives. Although participants identified with a single religion, they reported holding beliefs and engaging in practices associated with other religious traditions. To the best of our knowledge, this is the first study in Portugal to document such intrapersonal religious pluralism. This may be due to the qualitative nature of the study, which goes beyond declared religious affiliation and allows for a deeper exploration of shifts in the content of traditional beliefs, highlighting, in particular, that religious practice and religious identity are not always congruent (Hackett 2014).

Beliefs and reflections on substance use in the two contexts were different. In relation to the treatment aimed at people with SUD, the Portuguese participants expressed criticism of therapeutic communities (especially religious ones) the use of the Alcoholics Anonymous (A.A.) 12‐step model, the use of the same type of treatment for different individuals, the imposition of religious beliefs, and the deprivation of liberty, while in Brazil the participants did not express such criticism. This may be due to the fact that the Brazilian sample was derived from people undergoing treatment in a religious therapeutic community while the Portuguese sample comprised people who were in shelters, with unlimited access to the harm reduction approach. This suggests that the places that help people with SUD also tend to shape their perceptions about drugs and treatment, which can stimulate or inhibit reflection (Allen et al. 2019). Moreover, there is a body of research showing that the harm reduction approach tends to provide people with greater knowledge about drugs, encourage less prejudice against people who use drugs, and prompt reflections based on scientific data (Allen et al. 2019; Gallagher et al. 2019).

The high number of Portuguese participants who criticized such forms of treatment suggests that this may be a topic that should be explored in greater depth in future research. For example, it could be valuable to analyze whether people in harm reduction contexts might be more sensitive to reflections on forms of treatment than those in a more repressive context (such as the Brazilian sample). It is important to note that some of these comments made by the Portuguese participants are in line with current criticisms in surveys carried out on therapeutic communities (CRP 2017; IPEA 2017). Another possible explanation that may explain these differences between the two samples is the ages of the sample, which in Portugal included more older people with longer drug use. Greater experience of drug use and care and treatment services may have resulted in a more informed analysis of these issues. Finally, the reflection on the treatment itself and the less stereotypical view of people who use drugs found in the Portuguese sample may indicate that a treatment context not focused solely on abstinence can provide greater possibilities for reflection on the recovery process itself.

### 4.1 Limitations and Implications for Clinical Practice and Future Research

This study has some limitations. First, as an exploratory qualitative study it was not designed to provide a representative sample. Second, although both samples consisted of people in situations of social vulnerability, they are not directly comparable as the Brazilian sample was from a therapeutic community while the Portuguese sample was from a shelter. Although many Portuguese participants had previous experience of therapeutic communities, conducting the interviews in a setting that encouraged harm reduction may have led to much more critical responses.

Future research could investigate whether the concept of subjective protection also occurs among populations with stable housing or other health conditions, and whether it is simultaneously associated with both protective and risk factors, as observed in the present study. Furthermore, additional studies are needed on the relationship between drug use and religiosity in vulnerable populations, since the dimensions of religiosity observed in the general population may differ from or even contrast with those observed among homeless populations. These analyses can be done qualitatively, through ethnographies and interviews that delve into religious meanings and the persistence of risky behaviors, and quantitatively by analyzing, for example, whether beliefs in divine protection may be associated with greater risky behavior and, at the same time, a greater sense of unconditional divine security and purpose in life.

Our findings challenge conceptions that religiosity functions exclusively as a protective factor against drug use and as a supportive resource in the treatment of SUDs. They also contribute to the understanding of subjective protection as a complex dimension that may help clinicians recognize that religiosity can simultaneously operate as both a protective and a risk factor. The religiosity brought by patients can be incorporated into a holistic health assessment that respects individual beliefs and considers religiosity as a belief system integrated into preventive approaches and attitudes toward health. However, although religiosity may constitute a resource for strengthening healthcare, it should be addressed based on the content brought forward by the patient rather than on the professional's personal beliefs. The ability to differentiate patients' beliefs and values from one's own personal beliefs, while avoiding imposing the latter during care, should be part of professional training. An individualized approach and an understanding of how each individual constructs meaning from their religious experience may assist in the development of strategies aimed at reducing risk factors related to drug use. This approach can help professionals leverage diverse religious beliefs to promote health education and tailor harm reduction strategies. Clinical work may encompass both the reframing of personal religious beliefs that underlie risky behaviors (e.g., sharing needles or exposing oneself to transmissible diseases) and the development of individualized self‐care strategies, using religiosity itself as a resource already strengthened and rooted in each individual's life history.

## 5 Conclusion

In conclusion, in both samples, religiosity played a complex role, indicating that drug use and religiosity are more closely intertwined than previously assumed. These findings reveal a distinct dynamic in the relationship between religiosity and drug use, diverging from the prevailing perspectives in current research. Despite the differences and similarities in the meanings attributed to drug use and religiosity by participants from both countries, the proximal and interconnected nature of these two themes is evident in both contexts. Religiosity was used as a source of support, security, and meaning, whether alongside drug use, as a warning of the dangers of drug use, or independently. This insight may facilitate a deeper understanding of the role of religiosity in homeless individuals with SUDs.

## Author Contributions

**Leonardo Breno Martins:** investigation, writing – review and editing, formal analysis, methodology. **Wellington Zangari:** supervision, writing – review and editing. **Tassiane Cristine Santos de Paula:** methodology, writing – review and editing. **Camila Chagas:** conceptualization, investigation, funding acquisition, writing – original draft, methodology, supervision, formal analysis, writing – review and editing. **José Carlos Fernandes Galduróz:** supervision, writing – review and editing, methodology.

## Funding

This study is supported by a grant from the São Paulo Research Foundation (FAPESP); C.C. (PhD scholarship—2020/10234‐2 and 2021/13548‐0).

## Ethics Statement

This study was approved by the Ethics and Research Committee of the Federal University of São Paulo. Project No. 0931/2020.

## Consent

Informed consent was obtained from all individual participants included in the study.

## Conflicts of Interest

The authors declare no conflicts of interest.

## Supporting information


**Data S1:** Interview questions.

## Data Availability

The data that support the findings of this study are available from the corresponding author upon reasonable request.
