# Supplementary material for: Walking Together: A Cross‐Cultural Study of the Relationship Between Religiosity and Substance Use Disorder
Source: Nurs Health Sci. 2026 Aug 27;28(3):e70418. doi: 10.1111/nhs.70418 (PMC13519535; doi:10.1111/nhs.70418)
Supplement: Supplementary file 1 [file NHS-28-e70418-s001.docx]

**Interview questions**

Demographic Information: Age, education level, occupation, and number of children.

Information about drug use: Questions about the participant’s history of drug use.

Information about religious beliefs: Questions about the participant’s religious beliefs and whether these beliefs interact with their drug use.

1. Could you please tell me a little about your history with drug use?
2. At what age did you begin using _____ regularly (e.g., amount and frequency)?
3. Are you currently undergoing treatment for the use of which substance?
4. How long have you been receiving treatment here?
5. Have you undergone any other treatments in the past?
6. What is your religion or religious affiliation?
7. Were you raised with any religion?
8. How important is religion in your life?
9. Do you currently attend a church or other religious institution?
10. Did you attend a church or other religious institution in the past?
11. In your opinion, how does your religion view drug use? Do you agree or disagree with this view?
12. Closing question: Is there anything else you would like to discuss that has not already been covered?
